# Integrated Transcriptional Regulatory Network of Quorum Sensing, Replication Control, and SOS Response in *Dinoroseobacter shibae*

**DOI:** 10.3389/fmicb.2019.00803

**Published:** 2019-04-12

**Authors:** Sonja Koppenhöfer, Hui Wang, Maren Scharfe, Volkhard Kaever, Irene Wagner-Döbler, Jürgen Tomasch

**Affiliations:** ^1^Group Microbial Communication, Technical University of Braunschweig, Braunschweig, Germany; ^2^Institute for Chemistry and Biology of the Marine Environment, University of Oldenburg, Oldenburg, Germany; ^3^Group Genomic Analytics, Helmholtz Centre for Infection Research, Helmholtz Association of German Research Centers, Braunschweig, Germany; ^4^Research Core Unit Metabolomics, Institute of Pharmacology, Hannover Medical School, Hanover, Germany

**Keywords:** Roseobacter, quorum sensing, CtrA, LexA, gene transfer agent, c-di-GMP, chromosome location, replication

## Abstract

Quorum sensing (QS) coordinates population wide gene expression of bacterial species. Highly adaptive traits like gene transfer agents (GTA), morphological heterogeneity, type 4 secretion systems (T4SS), and flagella are QS controlled in *Dinoroseobacter shibae*, a Roseobacter model organism. Its QS regulatory network is integrated with the CtrA phosphorelay that controls cell division in alphaproteobacteria. To elucidate the network topology, we analyzed the transcriptional response of the QS-negative *D. shibae* strain Δ*luxI_1_* toward externally added autoinducer (AI) over a time period of 3 h. The signaling cascade is initiated by the CtrA phosphorelay, followed by the QS genes and other target genes, including the second messenger c-di-GMP, competence, flagella and pili. Identification of transcription factor binding sites in promoters of QS induced genes revealed the integration of QS, CtrA phosphorelay and the SOS stress response mediated by LexA. The concentration of regulatory genes located close to the origin or terminus of replication suggests that gene regulation and replication are tightly coupled. Indeed, addition of AI first stimulates and then represses replication. The restart of replication comes along with increased c-di-GMP levels. We propose a model in which QS induces replication followed by differentiation into GTA producing and non-producing cells. CtrA-activity is controlled by the c-di-GMP level, allowing some of the daughter cells to replicate again. The size of the GTA producing subpopulation is tightly controlled by QS via the AI Synthase LuxI_2_. Finally, induction of the SOS response allows for integration of GTA DNA into the host chromosome.

## Introduction

Microbial communication based on quorum sensing (QS) is a process whereby bacteria adjust their behavior depending on the cell density (Miller and Bassler, 2001). A QS system consists of one or more synthases that produce small diffusible molecules termed autoinducers (AI) which are released to the exterior of the cell. A minimum threshold of AI is sensed by corresponding receptors that thereupon adjust the gene expression directly or via signaling cascades. Multiple AI have been found, although oligopeptides and *N*-acyl-L-homoserine lactones (AHL) make up the majority of AI in Gram-positive and Gram-negative bacteria, respectively (Miller and Bassler, 2001; Papenfort and Bassler, 2016). Sensing of population density and integration of this information with environmental and intracellular signals allows for a coordinated decision-making process important for the production of public goods like antibiotics and exoenzymes, biofilm formation and dispersal, metabolic diversification of an isogenic population and self-destructive cooperation (Nadell et al., 2008).

A putative example for the latter process currently under debate (Redfield and Soucy, 2018) is horizontal gene transfer mediated by small virus-like particles. These so-called gene transfer agents (GTAs) are ubiquitous in *Rhodobacterales* and especially well-studied in *Rhodobacter capsulatus*. GTAs are considered to be descendants of a prophage that was integrated into an alphaproteobacterial ancestor and was brought under the control of the QS gene regulatory system of the bacterial host (Lang and Beatty, 2007).

*Rhodobacter capsulatus* and *Dinoroseobacter shibae* are the only two organisms known in which QS controls expression of the CtrA phosphorelay and its targets such as GTAs. This relay consists of a histidine kinase CckA, phosphotransferase ChpT and transcriptional regulator CtrA and is best known for its role in controlling the asymmetric cell division of *Caulobacter crescentus* (Laub et al., 2002) into dimorphic cells being either swarmer or stalked with pilus and flagellum or stalk and holdfast, respectively (Mignolet et al., 2018). In *R. capsulatus* CtrA regulates expression of genes for GTA synthesis, competence family proteins and motility (Leung et al., 2012, 2013; Mercer et al., 2012; Brimacombe et al., 2014). A physiological clue that is integrated in the downstream decision making process is DNA strand stress via the SOS response master regulator LexA (Kuchinski et al., 2016). The LexA repressor undergoes autoproteolysis upon interaction with a complex of RecA and single stranded DNA (ssDNA). In *R. capsulatus* LexA autoproteolysis releases the promoter of *cckA* and thereby affects the phosphorylation of CtrA and its regulated traits (Westbye et al., 2017b, 2018).

Quorum sensing systems are especially common in the diverse and widely distributed Roseobacter group of marine *Rhodobacteraceae* (Simon et al., 2017). Members of this group represent the predominant part of the prokaryotic community in algae blooms (Gonzalez and Moran, 1997; Segev et al., 2016) and *Dinoroseobacter sp*. have been found to dominate the bacterial community associated with the dinoflagellate *Ostreopsis* cf. *ovata* (Guidi et al., 2018). QS in the Roseobacter group is of special interest as algal blooms and biofilms are environments that are better suited for communication via diffusible molecules than the open ocean (Cude and Buchan, 2013).

The QS system of *D. shibae* is one of the best studied among the Roseobacters. It consists of three AHL synthases, two of them located on the chromosome (LuxI_1,2_) and one on the 86-kb plasmid (LuxI_3_), each of them in an operon with a putative LuxR-type transcriptional regulator (Patzelt et al., 2013). *D. shibae* produces long chain AHLs with varying side chain lengths (C14–C18) and modifications (Neumann et al., 2013; Wang et al., 2014b). While LuxI_1_ was identified to mainly synthesize AHLs with side chain length of 18 carbon atoms (C18en and C18dien HSL), LuxI_2_ and LuxI_3_ synthesize mainly AHLs with side chains of 14 carbon atoms (C14en and 3-oxo C14 HSL) (Neumann et al., 2013; Patzelt et al., 2013; Wang et al., 2014b). The exact structure of the AHLs synthesized in the original host *D. shibae* differs from that of the enzymes cloned in *E. coli;* experiments with purified AI synthases showed that this is caused by the type of fatty acids available (Ziesche et al., 2018).

The *D. shibae* QS system is organized hierarchically, as revealed by gene deletion analyses: Deletion of *luxI_1_* results in a QS null mutant which does not produce detectable levels of AHLs (Patzelt et al., 2013). The product of the LuxI_1_ synthase induces expression of the CtrA phosphorelay which induces expression of the *luxI_2_* and *luxI_3_* operons as well as QS target genes, including those for biosynthesis of flagella and GTAs. In contrast, the plasmid located type IV secretion systems (T4SS) are induced by QS but not as a part of the CtrA phosphorelay (Wang et al., 2014b). QS activation of the CtrA phosphorelay has been shown to affect the chromosome content of cells and their morphological differentiation (Patzelt et al., 2013; Wang et al., 2014b). The effects of the *luxI_1_* knockout could be complemented by external addition of a wide variety of long chain AHLs (Patzelt et al., 2013, 2016). While knockout of *luxI_1_* and CtrA phosphorelay genes resulted in the decrease of the expression of GTA genes, knockout of *luxI_2_* resulted in their overexpression (Tomasch et al., 2018).

In this study, we aimed to unravel the temporal sequence of gene regulation of the QS system in *D. shibae* by examination of the time series transcriptome of a QS null mutant (Δ*luxI_1_*) to external AI addition. By determining transcription factor binding sites *in silico* we further analyzed if in *D. shibae* the SOS response is incorporated via LexA into the decision making as it has been found for *R. capsulatus*. To understand the consequences of transcriptional activation on a physiological level we analyzed the influence of the added AI on the chromosome content on the single cell level by flow cytometry and determined the GTA major capsid protein (MCP) as well as the second messenger c-di-GMP. Since replication and QS have been shown to interact via the CtrA phosphorelay, we analyzed the chromosomal localization of the major transcriptional regulators in both *D. shibae* and *R. capsulatus* and found that most of them have conserved positions relative to the origin and terminus of replication. Based on this study we were able to develop a hypothetical model for the integration of QS, replication control, and SOS response in *D. shibae*.

## Materials and Methods

### Bacterial Strains, Growth Conditions, and AHLs

*D. shibae* strains DFL12*T* (DSM16493) Δ*luxI_2_* and Δ*luxI_1_* (Patzelt et al., 2013) were cultivated in the dark at 30°C and 160 rpm in half concentrated Marine Broth (MB) or the chemically defined artificial sea water media (SWM) supplemented with 5 mM succinate (Tomasch et al., 2011). To prepare 1 mM stock solutions of 3-oxo C14 HSL (Cayman Chemicals, Ann Arbor, MI, United States) this compound was dissolved in dimethylsulfoxid (DMSO) while the stock of 1 mM C18 dien HSL (Institute of Organic Chemistry, TU Braunschweig, Germany) was dissolved in dichloromethane (DCM). 3-oxo C14 HSL was chosen as inducing AHL for transcriptomics, as it is commercially available, while C18 dien HSL had to be synthesized by cooperation partners and was not available on a regular basis.

### Induction Conditions

Precultures were prepared by spreading glycerol stocks on ½ MB plates and used to inoculate 20 mL liquid media (SWM with 5 mM succinate) with a loopful of bacteria and incubated overnight. Main cultures were inoculated to an optical density at 600 nm (OD_600_) of ∼0.01 and grown to an OD_600_ of ∼0.22 before treatment with 500 nM 3-oxo-C14-HSL (Supplementary Figure S1, induced) or an equal volume of DMSO (non-induced control). The concentration of 500 nM has been shown to be the lowest concentration that fully restores the transcriptome of the *ΔluxI_1_* mutant for all tested AHLs (Patzelt et al., 2016). The onset of the logarithmic phase is reached at an OD_600_ of ∼0.2 (Supplementary Figure S2), at which point QS regulated traits have previously been shown to be already expressed (Patzelt et al., 2013). The influence of AHLs and DMSO on the growth rate of the cultures was determined in a separate experiment. It differed only slightly, thus no major differences for the effect of growth between AHL and DMSO treated cultures were expected (Supplementary Figure S3).

### Time Course Experiment and RNA Isolation

Three 120 mL main cultures of *D. shibae*Δ*luxI_1_* were inoculated from precultures as described above. After reaching the desired OD_600_ cultures were split into two cultures (induced and non-induced control) and incubated as described above. Before the split and at time points 10, 20, 40, 60, 120, and 180 min post-induction, two samples of 2 mL per treatment were withdrawn for transcriptomics and processed immediately. Additionally, 1 mL samples were withdrawn for flow cytometric analysis. The transcriptome samples were centrifuged (13,000 rpm, 1 min, room temperature), the supernatant was removed, and 500 μL Trizol reagent (Ambion, Life Technologies, Carlsbad, CA, United States) was added. Samples were snap frozen using liquid nitrogen and stored at -70°C. Isolation of RNA was performed as described (Wang et al., 2014a). RNA amount was determined using the NanoDrop spectrophotometer (Peqlab, Erlangen, Germany). The following samples were sequenced in three biological replicates: pre-induction, 10, 20, 40, 60, 120, and 180 min post-induction with AHL, 60 and 180 min after addition of the solvent DMSO (control).

### RNA Sequencing and Data Processing

Total RNA was treated with the RiboZero kit (Illumina, San Diego, CA, United States) to remove ribosomal RNA following the manufacturer’s protocol. Single end, strand specific cDNA libraries were prepared using the Scriptseq v2 RNA-Seq Library Preparation Kit (Illumina, San Diego, CA, United States) following the manufacturers protocol. For sequencing equal volumes of libraries (12 pM) were multiplexed on a single lane. Sequencing was done on the HiSeq 2500 (Illumina, San Diego, CA, United States) using TruSeq SBS Kit v3 – HS (Illumina, San Diego, CA, United States) for 50 cycles resulting in 50 bp reads. Image analysis and base calling were performed using the Illumina pipeline v 1.8 (Illumina, San Diego, CA, United States). The demultiplexed raw fastq-files were quality-controlled using the FASTQ-mcf suite^1^. Low quality bases (Phred-score <30) and Illumina adaptors were clipped. Reads were mapped to the reference genome using bowtie2 (Langmead and Salzberg, 2012) with default parameters for single end reads.

Ambiguously mapping reads were randomly distributed between all regions to which they could be assigned. The plasmids of *D. shibae* contain highly syntenic regions with strong sequence conservation. These include large parts of the 191 kb and 126 kb plasmids (Wagner-Döbler et al., 2010) as well as a transposable element with the *thiC* gene in center duplicated on three plasmids (Tomasch et al., 2018) As reads could not be assigned unambiguously we would otherwise loose information on the expression of these regions. None of these syntenic regions showed a change in expression in the conducted experiments.

The resulting sam-files were converted to indexed binary format and pile-up format using samtools (Li et al., 2009). The program featureCounts (Liao et al., 2014) was used to count the reads mapping to genes. Accession numbers of the reference genome of *D. shibae*: chromosome, 3.79 Mb (NC_009952.1); pDSHI01, 191 kb (NC_009955.1); pDSHI02, 153 kb (NC_009956.1); pDSHI03, 126 kb (NC_009957.1); pDSHI04, 86 kb (NC_009958.1); pDSHI05, 72 kb (NC_009959.1).

### Data Availability

Raw and processed RNA-sequencing data have been deposited at the gene expression omnibus database under accession number GSE122111^2^.

### Statistical Analysis

The quality of replicate samples was assessed by manual inspection of scatter plots and by calculating the Pearson correlation coefficient (Supplementary Figure S4). The R-package edgeR (Robinson et al., 2010) was used to identify genes with a significant change in expression. Differential expression was assessed separately for the AHL and DMSO treated samples. Only genes with a false discovery rate <0.05 and an absolute log^2^ fold-change >1 compared to time point *t* = 0 were considered. Additionally, genes that had an absolute log fold-change difference >1 between AHL treatment and DMSO treatment (control) at 60 min or 180 min were removed from the dataset (approximately 130 genes), as these genes likely had changed either due to addition of DMSO or as a result of cultivation time. The dataset was further manually checked for genes slightly below the chosen cut-off but likely not part of the QS response because they were part of an operon or gene cluster that also changed in expression during the control treatment. The strong down-regulation of photosynthesis-related genes in the later AHL-treated and DMSO-treated samples was most probably caused by light-exposure of the samples during sampling. We previously demonstrated the repressing effect of light on these genes (Tomasch et al., 2011). Differential expression of all 4,192 protein coding genes for all treatments can be found in Supplementary Table S1. The 243 genes considered as responding to the addition of AHLs and further analyzed can be found in Supplementary Table S2.

### Flow Cytometry

SybrGreen stains DNA stoichiometrically and is therefore suited for determination of the chromosome content of cells. In particular the two peaks often observed for slow growing bacteria allow to differentiate between pre- and post-replication cells (Marie et al., 1997). Samples of 1 mL were fixed using 80 μL 2% glutaraldehyde (Heidelberg, Germany) and analyzed using a BD FACS Canto flow cytometer (BD Biosciences, San Jose, CA, United States). Samples were diluted if necessary using PBS (8 g NaCl, 0.2 g KCl, 1.44 g Na_2_HPO_4_, 0.24 g KH_2_PO_4_, dissolved in 1 L distilled water, autoclaved and sterile filtered) and stained with 10 μL mL^-1^ SybrGreen I (Molecular Probes, Leiden, Netherlands). The signal was monitored using the FITC filter (excitation 488 nm, emission 519 nm).

### *In silico* Determination of Transcription Factor Binding Sites

CtrA transcription factor binding sites (TFBS) were determined using the matchPWM function of the R-package Biostrings as described before (Wang et al., 2014b). We used the position weight matrix (PWM) determined for several alphaproteobacteria (Brilli et al., 2010), but tested three different minimum matching scores of 85, 82.5, and 80% (Supplementary Table S3). After manual inspection of the obtained sequences we decided to use a cut-off of 82.5%. To determine LexA TFBS in *D. shibae* 80 alphaproteobacterial sequences with *in silico* determined LexA binding site were downloaded (Erill et al., 2004). These sequences had been obtained by comparison of alphaproteobacterial whole genome sequences with the known *E. coli* LexA binding sequence. Based on these sequences a position weight matrix (PWM) was generated and searched for in the *D. shibae* genome. Genes with a minimum matching score of 82.5% were kept and ordered to two groups, the GTTC consensus and GAAC consensus motifs (Supplementary Table S3). The sequence logo was obtained using the Weblogo service of the Computational Genomics Research Group (Crooks et al., 2004).

### Determination of the Origin and Terminus of Replication

Both origin (Ori) and terminus (Ter) of replication were obtained from the DoriC database (Luo et al., 2018) and confirmed using the GC skew. The GC skew describes the preference of guanine and thymine on the leading and adenine and cytosine on the lagging strand. Thereby, a switch between nucleotide preferences at the Ori and Ter has been observed (Lobry, 1996).

### Western Blots

Western blotting was performed to detect the MCP of GTAs. Main cultures of Δ*luxI*_1_ and Δ*luxI*_2_ had been prepared as described above. Strain Δ*luxI*_2_ was included as positive control as it is a GTA overproducing strain (Tomasch et al., 2018). Δ*luxI*_1_ was treated with 3-oxo C14, C18dien HSL or DMSO as negative control. A final concentration of 500 nM was used unless indicated otherwise. Samples of 400 μL were withdrawn, pelleted by centrifugation (17,000 × *g*, 2 min) and resuspended in 400 μL TE buffer. 5 μL of each sample was mixed with 6× Laemmli sample buffer. Before Western blotting, samples were heated (98°C, 5 min) and centrifuged (3,000 rpm and 2 min). SDS-PAGE and immunoblotting were performed as described before (Fu et al., 2010). However, instead of 10% polyacrylamide gels, 12% gels were used for protein separation and the antibodies were added at concentrations of 1:1000. The primary antibody (AS08365, Agrisera AB), detecting the GTA MCP (Dshi_2174) was incubated at 4°C overnight and the AP conjugated secondary antibody (AS09607, Agrisera AB) was incubated for 1 h. For chemiluminescent detection, the membrane was incubated in 1% alkali-soluble casein (Merck Millipore) utilized as blocking substance until visualization. For luminescence induction the membrane was then covered with CDP-Star AP Substrate (Merck Millipore) and signals visualized using the imaging system Las-3000 (FujiFilm Europe GmbH).

### Extraction and Quantification of C-di-GMP

Cultures were grown to the desired OD_600_, 5 mL were withdrawn, and samples were centrifuged (20 min, 4°C, 2,500 × *g*). The supernatant was discarded, and the pellet resuspended in 500 μL SWM. The sample was centrifuged again but the remaining pellet was resuspended in 300 μL solvent (acetonitrile/methanol/water 2:2:1). Incubation on ice for 15 min and heating at 95°C for 10 min were followed by cooling down on ice. After further centrifugation (10 min, 4°C, 20,800 × *g*) the pellet was resuspended in 200 μL solvent and the supernatant stored at -20°C overnight for allowing optimal protein precipitation. The pellet was resolved in 200 μL solvent (acetonitrile/methanol/water 2:2:1) (without heating to 95°C again), and this step was repeated twice. The final pellet was used for determination of total protein concentration applying the bicinchoninic acid (BCA) method as described below. The supernatant of overnight protein precipitation was centrifuged for 10 min at 4°C, 20,800 × *g* and the resulting supernatant stored at -70°C. C-di-GMP was quantified in this supernatant using mass spectrometry as described before (Bähre and Kaever, 2017).

### Determination of Protein Concentration

The total protein concentration was determined using the BCA assay kit (Lot P120456, Serva) according to the manufacturer’s protocol. Pellets from 1 mL cell culture were resuspended in 800 μL 0.1 M NaOH and heated for 15 min at 95°C. Successful lysis of the cells was controlled by microscopy. Cell debris was removed by centrifugation (10 min, 4°C, 20,800 × *g*) and 100 μL supernatant was transferred to 2 mL BCA working solution and incubated at 60°C for 15 min. After cooling to room temperature, the OD was determined at 562 nm photometrically (Ultrospec 3100 pro).

## Results

We followed the transcriptional response of the QS null mutant *D. shibae*Δ*luxI_1_* toward an externally added AHL for 3 h. In order to discriminate between fast and slow responding genes, the first hour post-induction was densely sampled. To induce QS, we spiked the culture with 500 nM 3-oxo C14 HSL. This AHL is the second most abundant AHL synthesized by *D. shibae* (Ziesche et al., 2018) and it is the product of the synthases LuxI_2_ or LuxI_3_ which are under the control of LuxI_1_ (Patzelt et al., 2013). Previously we showed that various long chain AHLs of *D. shibae*, including the most abundant AHL, C18 dien HSL, as well as 3-oxo C14 HSL fully restore the effects of the *luxI_1_* deletion on the transcriptional and physiological level (Patzelt et al., 2016).

### Time Series Transcriptomic Response Toward AHL Addition

**FIGURE 1:**
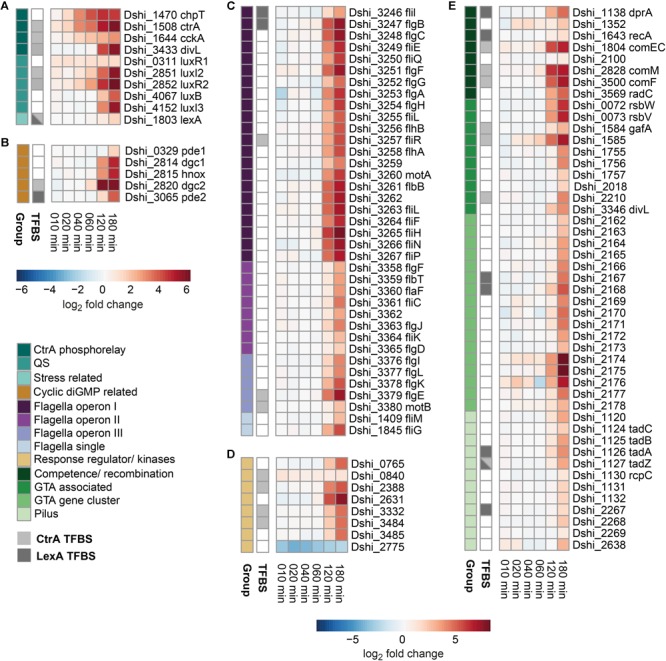
Time resolved transcriptomic analysis of a *D. shibae* quorum sensing null mutant response toward 3-oxo C14 HSL addition. *D. shibae* Δ*luxI_1_* cells were induced by addition of either 500 nM AHL or DMSO. At multiple time points samples were withdrawn and the differential gene expression evaluated and displayed in this heatmap. Regulated genes were organized into the color indicated categories displayed in multiple heatmaps **(A–E)**. Transcription factor binding sites (TFBS) for CtrA and LexA were obtained by matching a position weight matrix based on known binding sites in other alphaproteobacteria on the *D. shibae* genome.

The time series analysis of the transcriptome up to 3 h post induction (Supplementary Table S1), revealed 243 genes with significant change of expression compared to *t* = 0 (before induction), of which 235 were located on the chromosome (Supplementary Table S2). Only 49 genes were downregulated, most strongly the putative transcription factor Dshi_2775. The alginate-biosynthesis operon provided a rare example of transient activation within the first hour post induction. Below we focus on potential targets activated by the CtrA phosphorelay and genetic modules to which we could assign clear functions using comparative genomics (Supplementary Table S4). The expression profiles of these 107 selected genes are displayed in Figure 1.

### Quorum Sensing and CtrA Phosphorelay

In Figure 1A genes of the CtrA phosphorelay (*ctrA, chpT, cckA*), AHL synthases (*luxI_2_, luxI_3_*), the corresponding transcriptional regulators (*luxR_1_* and *luxR_2_, luxB*), the cell division protein *divL* (included due to its CckA modulating effect in *Caulobacter*) and the response regulator *lexA* are shown. A clear timing of transcription initiation could be observed (Supplementary Figure S5): *CtrA* and *chpT* showed an immediate sharp increase in expression while *cckA* expression increased with a less steep slope. While *chpT* reached a maximum after 60 min, transcriptional activation of the other two genes slowed down after 40 min but continued to increase. The QS genes *luxI_2_/luxR_2_* started to increase 20 min after AHL addition, and *luxI_3_/luxB* transcription increased more than 60 min after stimulation with AI. Interestingly, the regulator LuxR_1_, located adjacent to the deleted master synthase LuxI_1_, was induced already 10 min after AHL-addition and remained at this level throughout the experiment. The genes *divL* and *lexA* increased in expression at 2 and 3 h, respectively (Figure 1A).

### Enzymes Involved in C-di-GMP Turnover

We found all recently characterized enzymes involved in c-di-GMP turnover of *D. shibae* (Bedrunka et al., 2018) to be induced by AHL addition (Figure 1B). C-di-GMP is synthesized through condensation by diguanylate cyclases (DGC) and hydrolyzed by phosphodiesterases (PDE). Most organisms possess multiple DGC/PDEs that play a role in various processes (Hengge et al., 2015). *D. shibae* has two PDE, two DGC and one H-NOX gene which inhibits the adjacent *dgc1.* Our data revealed a precise timing in the induction of these genes by QS: *dgc2* is expressed first from 1 h onward. *Dgc1* and *hnox* expression increased at 2 h post-induction while the hydrolyzing enzymes *pde2* and *pde1* are activated from 2 and 3 h onward, respectively (Figure 1B). The actual metabolite formation was proven (see below).

### Flagella Gene Cluster

The *D. shibae* flagellum is built from three gene clusters (gene cluster I–III) and two single genes (Figure 1C and Supplementary Table S5). Gene cluster I comprises 22 genes of the flagella basal body, biosynthesis and motor proteins. Gene cluster II comprises eight genes of the flagella regulatory system, flagellin and flagella hook protein. Gene cluster III comprises five genes of hook proteins and chemotaxis system. FliM and FliG are separately located and are known for their roles in the rotor behavior of flagella. Here, we found cluster I to increase in expression at 2 h post-induction while gene cluster II is activated at 3 h. Gene cluster III and the single genes are showing both patterns, activation at 2 or 3 h, depending on the gene. Interestingly the timing of transcriptional activation corresponds to the order of flagella construction, from the components located at the inner membrane to the extracellular parts. A similar expression pattern, dividing the flagella genes in early, middle, and late expressed genes depending on their location, has been described for multiple organism including *Escherichia coli* and *Salmonella enterica* (Chilcott and Hughes, 2000).

### Other Regulators and Kinases

During the annotation process multiple regulators and kinases potentially involved in signal transduction cascades were identified (Supplementary Table S4). These genes could not be assigned to specific regulatory networks but showed strong differential expression (Figure 1D). Notably, some of these regulatory genes are located near the origin of replication (see below).

### Genes Associated With Competence, GTA, and Tad Pilus

DprA, RecA, and the Com family proteins are generally known for their role in competence (DNA uptake from the environment). In *R. capsulatus* their role in GTA uptake by recipient cells has been shown by gene deletion experiments (Brimacombe et al., 2015). Here, the Com proteins showed already strong activation 120 min post-induction with AHLs.

While Dshi_1585 expression showed an increase already after 40 min, the neighboring gene Dshi_1584, the homolog of the recently identified *R. capsulatus* direct activator of GTA gene cluster, *gafA* (Fogg, 2019) started slightly to increase in expression 2 h post-induction. Part of the GTA gene cluster (including Dshi_2174, encoding the MCP) as well as the anti-sigma factor/antagonist pair rsbW/V started to increase in expression at 120 min while the remaining genes of the GTA gene cluster increased in expression 180 min post-induction (Figure 1E). We were further able to annotate multiple GTA associated genes that are involved in GTA synthesis but are not located in the GTA gene cluster (Supplementary Table S4). Notably, the genes in the more upstream part of the GTA gene cluster showed a weak but significant transient increase in expression 20 min post-induction.

Genes originally annotated as type II secretion system were found to be closely related to the Tad pilus secretion system (Supplementary Table S4). This system is encoded by two gene clusters (Dshi_1117-1132, 2267-2269) and a single gene (Dshi_2638). Some of these genes were not identified in the processed transcriptomic data but still showed similar activation behavior, however, below the cut off criteria. The pilus subgroup is the last to be upregulated, at 180 min at a low, but increasing level which might be the reason for the elimination of genes of this cluster from the processed data.

### Putative Transcription Factor Binding Sites for CtrA and LexA

Now we asked how CtrA was involved in the regulation of the examined traits. Therefore we determined transcription factor binding sites (TFBS) *in silico* with a minimum score of 82.5% identity of the PWM (Supplementary Figure S6) and indicated them next to the transcriptomic data of each gene (Figure 1). We predicted TFBS in the promoter regions of *ctrA* and *cckA*. This indicates a feedback loop as CtrA switches into its active version after receiving a phosphate residue from CckA. The phosphorylated CtrA then increases transcription of *ctrA* and *cckA*, resulting in an even higher concentration of active CtrA. Furthermore *luxI_2_/R_2_* and the flagella (Dshi_3257/3379/3380) show CtrA binding sites. The integration of the QS system and CtrA phosphorelay has been known before as well as the regulation of the flagella by QS (Wang et al., 2014b). Additionally, we found putative TFBS in the promoters of the response regulator *lexA*, as well as in the promoter of *dgc2* coding for a c-di-GMP synthesizing enzyme. All three Com-family proteins, possibly involved in uptake of GTA DNA, as well as GTA associated genes (*gafA*, Dshi_1585/2210), the Tad pilus (Dshi_1127) and four regulator/kinase genes (Dshi_0840/2388/3332/3484) had TFBS for CtrA.

To determine if the SOS response mediated by LexA is integrated into this network, we identified TFBS for LexA *in silico*. We found putative binding sites in the promoters of *lexA* itself, in *pde2* encoding the c-di-GMP hydrolyzing enzyme, flagella genes (Dshi_3246/3247), the recombination genes *dprA* and *recA*, the GTA gene cluster (Dshi_2167/2168) and the pilus (Dshi_1126/1127/2267). In contrast to *R. capsulatus*, a binding site in the promoter of *cckA* could not be found. Additionally, the *in silico* determined LexA binding sites show no binding of any promoters of QS operons. However, LexA regulated traits overlap with CtrA regulated traits in multiple cases. TFBS for both regulators were found in the promotors of *lexA* itself and Dshi_1127 (Tad pilus), flagella, and c-di-GMP.

### Chromosomal Location of QS Regulators

**FIGURE 2:**
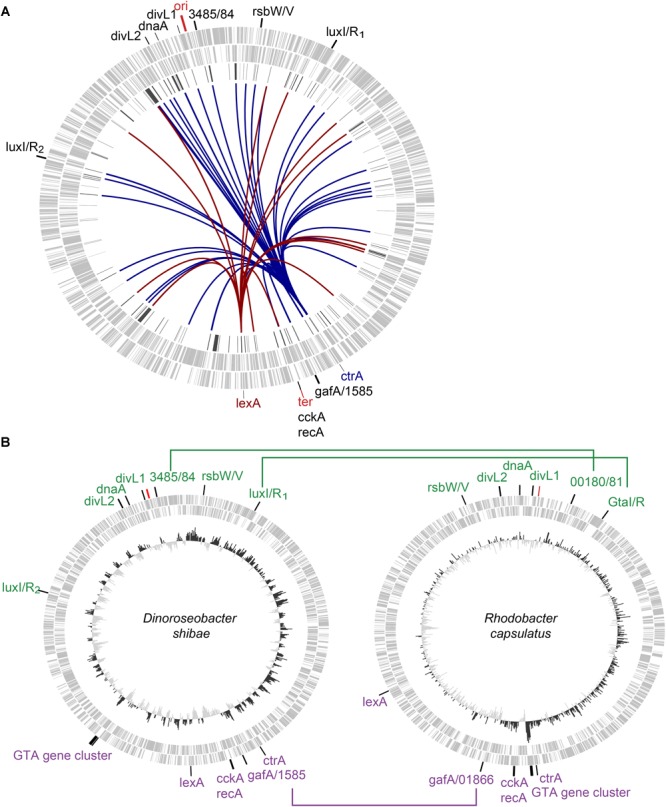
Location of selected regulatory genes on the chromosome of *D. shibae* **(A)** and *Rhodobacter capsulatus* **(B)**. Light gray circles indicate the chromosome strands (out = plus, in = minus). Position of origin of replication (*ori*) and terminus (*ter*), determined with website Dori are indicated. Chromosomal locations of genes with a potential or proven role in QS regulation are indicated. **(A)** The dark gray circle represents the QS regulated genes. Position transcriptional regulators *lexA* and *ctrA* are labeled and color indicated. The arcs connect LexA (red) and CtrA (blue) to their binding sites at respective locations. **(B)** *Ori*- and *ter*-located genes in both organisms are highlighted in green and purple, respectively. Orthologs genes with locus tag annotation are connected by lines. The inner circle shows the GC skew.

Next, we determined the chromosomal localization of genes and regulators of the *D. shibae* QS system, especially relative to the Ori and Ter regions (Figure 2A) and compared them to the chromosomal location of homologous regulators in *R. capsulatus* (Figure 2B).

The genes encoding the two synthases LuxI_1_ and LuxI_2_ and their corresponding receptors LuxR_1_ and LuxR_2_ (Dshi_0311/0312/2851/2852) are located symmetrically close to the *ori*.

By contrast, the major transcriptional regulators CtrA and LexA are symmetrically positioned around the terminus (Ter) (Figure 2A). The analysis of TFBS had already shown that they most likely regulate a multitude of QS genes directly. Here, blue and red lines visualize the even distribution of their regulated genes throughout the chromosome. *CckA/recA* are located directly at the Ter (Supplementary Figure S11). Adjacent to *ctrA*, the GTA regulators *gafA*/Dshi_1585 are located. *RsbW* and *rsbV* (Dshi_0073/0072) are homologs of the *R. capsulatus* sigma-factor antagonist and its repressor which control GTA gene expression (Mercer and Lang, 2014) and they are located between *luxI_1_* and the *ori*. Directly next to the *ori* the genes Dshi_3485/3484 are located. Dshi_3485 encodes a serine phosphatase domain that is similar to that of stage II sporulation protein E known for its role in septum formation during sporulation in *Bacillus* (Carniol et al., 2005). It further contains a signal receiver REC-domain to detect signals from the sensor domain of a two component system. Dshi_3484 has a Hpt (histidine phosphotransferase) domain and is possibly interacting with Dshi_3485 in a two component system but has not been assigned to a regulatory context in *D. shibae* here.

Two other genes, Dshi_3346 and 3433, are also located close to the *ori*. Both genes are considered DivL proteins after InterPro domain search that revealed the location of two PAS-domains in the CDS of Dshi_3433. Dshi_3346 was identified to contain one PAS domain, a histidine kinase and a signal transduction response regulator receiver domain of the CheY-like superfamily. For comparison, *R. capsulatus* SB 1003 *divL* (RCAP_rcc00042) contains one PAS-domain and *Caulobacter vibrioides* NA1000 *divL* (CCNA_03598) encodes two PAS-domains and a non-functional histidine kinase domain as identified by InterPro and Westbye et al. (2018). In *R. capsulatus* DivL regulates CtrA and GTA and in *C. crescentus* it affects cell cycle regulation via CtrA (Purcell et al., 2008; Westbye et al., 2018). Between the *divL* genes *dnaA* (Dshi_3373) was identified.

The location of homologous genes in *R. capsulatus* shows a very similar pattern (Figure 2B). *R. capsulatus* has one QS operon, the *gtaI/R* system, which is found in a similar chromosomal position as *luxI/R1*. Also, *divL1, divL2, dnaA* and *ctrA* can be found at the respective similar positions. The distance to the *ori*/*ter* is similar for *rsbW/V* and rcc01866/rcc01865. Only rcc_00180/1, *lexA* and *cckA/recA* are located further away from *ori*/*ter* compared to the location of the corresponding homologs in *D. shibae.*

### AHL Induced Physiological Changes

**FIGURE 3:**
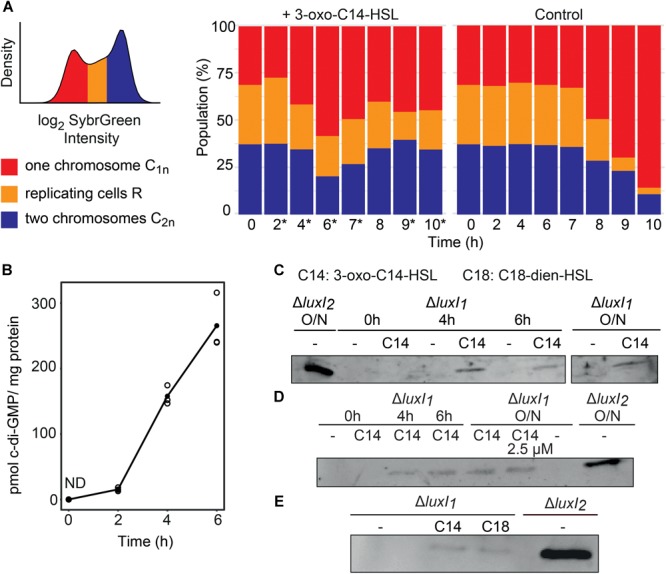
Changes in physiological traits expressed by *D. shibae* Δ*luxI_1_* in response to external AHL addition. **(A)** Flow cytometrical determination of the chromosome content by measuring fluorescence intensities of SybrGreen stained cells. The distribution of log_2_ SybrGreen intensity in the cells (**A**, left side) shows two peaks C_1n_ (red) and C_2n_ (blue) that represent one and two chromosome equivalents, respectively. Cells undergoing replication (yellow) are located in between. The distribution of the relative chromosome content in the population until 10 h post-induction is displayed for cultures induced by 3-oxo C14 HSL and the control cultures (**A**, right side). Asterisks indicate significant differences in the proportion of replicating cells between AHL and DMSO treated samples determined with a two-sided *t*-test. The experiment has been reproduced four times with three replicates each (Supplementary Figures S7–S10). **(B)** Second messenger c-di-GMP concentration post-induction with 500 nM 3-oxo C14 HSL. Concentrations in pmol c-di-GMP per mg protein were determined for 6 h post-induction by HPLC. ND, not detectable. **(C–E)** Western blot detection of the GTA major capsid protein in cell extracts of Δ*luxI_1_* induced with 3-oxo C14 (C14) or C18 dien HSL (C18) or non-induced as indicated. **(C)** Cell extracts at 0, 4, 6 h post-induction and after overnight (O/N) cultivation with 3-oxo C14 HSL and of Δ*luxI_2_* as positive control. **(D)** Major capsid detection in cell extracts of cultures induced with 500 nM or 2.5 μM AHL and incubated overnight. **(E)** Induction with 3-oxo C14 or C18 dien HSL.

Using SybrGreen staining of DNA, it was previously shown that QS signaling in *D. shibae* results in a subpopulation of elongated cells with increased chromosome content (Patzelt et al., 2013, 2016). This method allows for differentiation of cells according to their chromosome content (one or two chromosomes shown as peaks with replicating cells in between). Here, we monitored changes in chromosome content over a period of 10 h post-induction by flow cytometry (Figure 3A and Supplementary Figures S7–S10). At *t* = 0 the chromosome content showed two peaks reflecting populations before (one chromosome, C_1_) and after replication (two chromosomes, C_2_). Between the two peaks a population of cells undergoing replication (R) was found. Under these experimental conditions, our data consistently did not indicate accumulation of more than two chromosome equivalents in a sub-population, and population heterogeneity was not observed (see section “Discussion”).

All replicates of the control showed a similar relative abundance of all three populations (C_1_, R, C_2_) until 7 h. Then, a strong reduction of the replicating and post-replication populations (R, C_2_) was observed, indicating the onset of stationary phase. In contrast, AHL treated cultures increased the replicating fraction at 2 h post-induction significantly. Then the replicating and post-replication populations were reduced from 3 h until 6 h and increased again until the end of the experiment without indication of the onset of the stationary phase. The data show that the AHL directly influenced the pattern of chromosomal replication while we observed balanced growth in the control.

As we found all enzymes involved in c-di-GMP turnover significantly expressed in our time series transcriptomic data (Figure 1C), we were interested in how the expression of these enzymes affected the synthesis of the actual metabolite. Therefore, we determined the metabolite concentration in relation to the total protein concentration during a 6-h period post-induction with AHL (Figure 3B). No c-di-GMP was detected before induction. Between 2 and 6 h the concentration increased from 14.98 to 240.76 pmol c-di-GMP/mg protein. A decrease or plateau formation due to the onset of expression of c-di-GMP hydrolyzing enzymes (*pde*) was not observed.

Next, we analyzed actual GTA production on the protein level. The MCP was already detected by Western blotting at 4 h post-induction with 500 nM 3-oxo C14 HSL. The obtained band was much weaker than that of the Δ*luxI_2_*-strain used as a control (Figure 3C). We then tested if we could increase GTA production by modifying experimental conditions. First, we prolonged the incubation time with AI (overnight) (Figure 3C), second, we increased the AI concentration to 2.5 μM (Figure 3D) and finally we applied C18-dien-HSL, the main AI of *D. shibae* (Figure 3E), but the amount of MCP remained low. We have recently shown that GTA synthesis is suppressed by LuxI_2_ (Tomasch et al., 2018), which is confirmed by these experiments. Our data suggest that only a small fraction of the population synthesizes GTA upon AI induction.

## Discussion

### The Expanded QS Regulon of *D. shibae*

To study the QS response of *D. shibae* we used time series transcriptomics and *in silico* determination of transcription factor binding sites (TFBS). Multiple QS controlled traits had been identified before. Among these are GTAs, flagella, LuxI_2_ and LuxI_3_ synthases (Wang et al., 2014b), the T4SS and chromosome replication (Patzelt et al., 2016). While previous analyses relied on the comparison of single gene knock-outs during the mid-exponential phase of growth, here we analyzed the global transcriptomic response of the culture during the first 3 h after stimulation with an externally added AI to unravel the timing of events and the integration between the different signaling circuits. Up-regulation of T4SS was not observed in our experimental set-up, suggesting that it occurs at the late exponential phase of growth.

**FIGURE 4:**
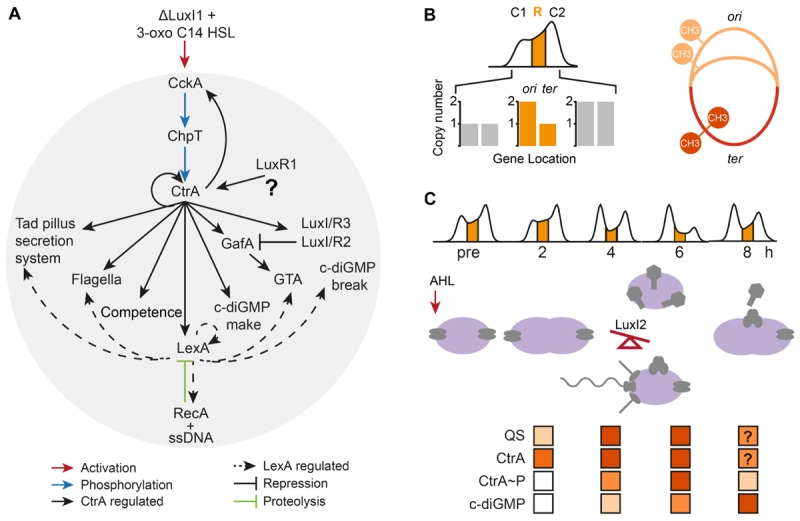
Quorum sensing regulatory system in *D. shibae*. **(A)** 3-oxo C14 HSL might be detected by the histidine kinase CckA or another sensor protein (red arrow) resulting in a phosphorylation cascade activating CtrA (blue) and subsequently its target genes (continuous arrows). LuxI_2_ might restrict GTA expression to a subpopulation (inhibiting arrow). The SOS stress response regulator LexA and CtrA (dashed arrows) regulate overlapping traits. The activation of LexA by RecA binding to ssDNA induces autoproteolysis of the stress regulator (green). **(B)** Possible impact of QS induced replication within the first 2 h post-induction on *ori* and *ter* located genes. Copy-number differences might alter the transcriptional balance of QS regulators close to origin and terminus of replication (left panel). Hemi-methylation during replication might impact transcription of CtrA-target genes (right panel). **(C)** Proposed model for the early response of *D. shibae* toward QS signaling: AHL detection results in fast activation of CtrA followed by an induction of replication as well as QS and c-di-GMP synthesizing genes. The increase in CtrA and its phosphorylation by CckA leads to repression of replication and initiates cell division and differentiation. The autoinducer synthase LuxI_2_ is the key factor in the decision making between GTA producing self-sacrificing cells and cells which form flagella, pili, and become competent for uptake of GTA-DNA between 2 and 4 h after induction. Following the stop of replication for 6 h, increasing c-di-GMP levels induce CckA mediated dephosphorylation of CtrA in some cells that start replicating again. The late induction of the SOS response could stimulate recombination of transferred DNA.

Our analyses of CtrA, LexA, and QS in *D. shibae* lead to the model shown in Figure 4A. Based on comparative genomics we were able to add novel traits to the known QS regulatory network, namely synthesis of c-di-GMP, Tad pilus, and competence related proteins, which are discussed in more detail below. Detection of the AI occurs most likely at the cell surface, by an unknown receptor, resulting in simultaneous up-regulation of the intracellular transcriptional regulators CtrA and LuxR_1_ already 10 min after addition of the AI. Downstream regulation occurs by overlapping TFBS for CtrA and QS resulting in synthesis of flagella, Tad pilus and competence, while c-di-GMP is regulated by CtrA and LexA. The SOS response regulator LexA is controlled directly by CtrA, and it modulates expression of Tad, flagella, c-di-GMP breakdown and LuxR_2_. Population heterogeneity is likely for all of these traits.

Enzymes that synthesize and hydrolyse the second messenger c-di-GMP were found to be QS controlled and to be regulated by CtrA and LexA, respectively. The c-di-GMP level in the cell is modulated in response to multiple environmental signals (QS, light, ppGpp) and influences expression and activity of traits such as T4SS, Tad pili, flagella, GTA, chromosome replication and cell morphogenesis in *Bartonella* sp., *V. cholerae, R. capsulatus* and *C. crescentus* (Boutte et al., 2012; Laverde-gomez et al., 2014; Lori et al., 2015; Québatte et al., 2017; Singh et al., 2017). In particular, effects of c-di-GMP on flagella motility have been described (Armitage and Berry, 2010; Koushik et al., 2010). Regulating flagella activity by c-di-GMP would be in accordance with the simultaneous upregulation of flagella transcription and c-di-GMP levels at 2 h after induction. However, homologs of the involved regulators of other species (YcgR or DgrA) or any other gene with c-di-GMP binding PilZ domain could not be identified. The average swimming velocity of *D. shibae* on SWM liquid medium with succinate as a carbon source has been determined to be 1.59 μm/s (Mann et al., 2016). Only a subpopulation of the cells carries flagella (Patzelt et al., 2013). Flagella might additionally have a role in virulence factor secretion and biofilm formation (Soutourina and Bertin, 2003).

Moreover, we found the competence (*comEC/comM/comF*) and recombination (*dprA/recA*) systems to be regulated by CtrA and LexA, respectively. These genes are especially interesting in relation to the transfer of dsDNA of GTAs into the recipient cell and integration into its genome. In *Rhodobacter* GTAs inject dsDNA through the outer membrane into the periplasm and the dsDNA is transferred from the periplasm into the cytoplasm via the Com proteins ComEC, ComF, and ComM, and recombination is assumed to be conducted by DprA and RecA (Brimacombe et al., 2015). A similar uptake mechanism is probably also working in *D. shibae*. The Com protein family is also used by naturally competent strains for incorporation of naked DNA from the environment. Even though binding by DprA in the cytoplasm generally occurs on ssDNA, additional binding to dsDNA has been determined as well. It has been argued that organisms expressing ComM are expressing a DprA domain that allows binding of dsDNA (Brimacombe et al., 2015).

We also found most of the homologs to the *R. capsulatus “*multilocus genome”(Hynes et al., 2016) to be induced by QS. Among them is the sigma factor antagonist and its repressor (Dshi_0072/0073, *rsbW/rsbV*) which are involved in *R. capsulatus* GTA regulation (Mercer and Lang, 2014). Dshi_1584 is the homolog of *gafA* that has been recently identified as the direct activator of GTAs (Fogg, 2019) and Dshi_1585 is a homolog of rcc01866, a gene involved in the lytic release of GTAs (Westbye et al., 2017b). Also involved in GTA release are Dshi_1756/1757 which represent an endolysin/holin system (Westbye et al., 2013). The homologs of Dshi_2018/2162 in *R. capsulatus* belong to tail fibers (Brimacombe et al., 2013) and Dshi_3346 corresponds to *divL* which regulates CtrA in *Rhodobacter* (Supplementary Table S4) (Westbye et al., 2018).

We further identified genes of the *tad* pilus secretion system that revealed the sequence *tad Z/A/B/C* which have been annotated as *cpaE/F/G/H* in *C. crescentus* (Mignolet et al., 2018). This is in accordance with genome comparisons which discovered a corresponding, common, strongly conserved locus within the Roseobacter group (*cpaBC-ompA-cpaEF-tadBC*) (Slightom and Buchan, 2009).

The Tad (**t**ight **ad**herence) secretion system has first been described in *Actinobacillus* (Motherway et al., 2011) and is widely distributed in Archaea, Proteobacteria and Actinobacteria. For *Bifidobacterium* sp. colonizing the gastrointestinal tract it was found that these pili attach to carbohydrates of glycoreceptors at the host surface during the first steps of colonization and therefore sequence differences of *tad* genes might determine host specificity (Motherway et al., 2011). *D. shibae* was isolated from *Prorocentrum lima* and *Alexandrium ostenfeldii* (Biebl et al., 2005) and interactions with various microalgae have been observed (Wagner-Döbler et al., 2010; Wang et al., 2014a). Genes of the QS response including c-di-GMP and *tad* genes transiently increase in expression during later phases of co-cultivation (Wang et al., 2015). Thus, these traits might contribute to the interaction with the algal host.

### Integration of the LexA Based Stress Response Into the CtrA and QS Network

We found that CtrA regulates LexA due to the presence of *in silico* determined TFBS and saw a differential *lexA* expression upon AHL stimulation. Furthermore, LexA most likely interferes with almost all CtrA regulated traits. We found putative LexA TFBS in promoters of the newly identified Tad pilus, DNA uptake and recombination machinery, flagella, *lexA* itself, and c-di-GMP (Figure 4A). A regulation of LexA by itself through a feedback loop is common and has been found, e.g., in *S. meliloti* and *C. crescentus* (Erill et al., 2004). In *E. coli* LexA plays a key role in heterogeneity by transcriptional regulation of DNA repair, type III secretion system, prophages and toxins (Mellies et al., 2007; Kamenšek et al., 2010). A similar regulation has been suggested for *Pseudomonas aeruginosa, Salmonella* and *Bacillus subtilis* (Kamenšek et al., 2010).

The stress response regulon in alpha- and gammaproteobacteria was found to include sets of core proteins among which are LexA, RecA and ComM, e.g., in *C. crescentus* and *S. meliloti* (Erill et al., 2004). The DNA uptake machinery which has been suggested to take up GTA delivered DNA from the periplasm (Westbye et al., 2017a) contains LexA and CtrA regulated genes (Figure 1E). While Com family protein mediated uptake of DNA through the inner membrane is controlled by CtrA, recombination by *dprA* and *recA* is LexA regulated. Similarly, c-di-GMP synthesis is controlled by CtrA and break down via LexA. In both cases, CtrA and LexA control genes needed during successive stages of the mentioned processes. In contrast to *R. capsulatus* (Kuchinski et al., 2016), in *D. shibae* the promoter of *cckA* has no LexA binding site while a CtrA TFBS was identified *in silico*. These data show that regulation by CtrA and LexA is highly similar between *R. capsulatus* and *D. shibae.* Nonetheless, the missing LexA binding site in the promoter of *cckA* – one of the key genes of the CtrA phosphorelay – might cause significant regulatory differences.

### Timing of QS Activation and Replication

We monitored the chromosome dynamics in response to AI addition using flow cytometry. Four independent experiments revealed an increased number of replicating cells 2 h post-induction, followed by a cell division stop, where cells with one chromosome accumulated and the number of replicating cells and cells with two chromosome equivalents was reduced. After 3 h, the replicating subpopulation increased again (Figure 3A and Supplementary Figures S7–S10).

We had previously shown that QS causes morphological heterogeneity in *D. shibae* with a subpopulation of enlarged cells whose division is inhibited and which contain several chromosomes per cell (Patzelt et al., 2013). This response is mediated by the CtrA phosphorelay (Wang et al., 2014b). Here, we did not see cells with more than two chromosomes or morphological population heterogeneity. Instead we saw shifts in the fraction of replicating cells. Differences in the experimental setup help to explain these apparent discrepancies. In previous experiments, the AHL was added at the beginning of growth and cells were harvested in the mid-exponential phase, thus they grew for approximately 10 h after AI induction (Patzelt et al., 2013, 2016; Wang et al., 2014b). Furthermore, the OD_600_ at AHL induction was 0.01, thus only 5% of the OD_600_ at which the culture was induced in the current experiment. Consequently, in the previously published experiments the population was exposed to higher concentrations of AHL per cell and for a much longer time which might lead to a much stronger physiological response or even an overshooting of the QS system which might lock the cells in the early replicative state (2 h post-induction in the current experiment).

The observed successive increase and decrease of the fraction of replicating cells in response to the AI might affect the regulation of gene expression in multiple ways: Replication can influence the transcript number of regulatory genes located at the *ori* and *ter*, respectively (Figure 4B). As exemplified in the left panel, genes close to *ori* are present in higher copy number in replicating cells, their number doubles with each replication initiation (Schmid and Roth, 1987; Sousa et al., 1997). For example, in *B. subtilis* spore formation is regulated by a two-component system, in which the histidine kinase KinA is located close to *ter*, while the cognate response regulator Spo0F is situated at the *ori*; this leads to pulsing increasing levels of Spo0F with every cell cycle and only if a certain KinA level is reached after several rounds of cell division, spore formation is initiated in a subpopulation. In such a way spore formation is inhibited if replication has not been completed (Lazazzera and Hughes, 2015; Narula et al., 2015). In our study we found that QS controlled regulators (DivL1/2, RsbW/RsbV, DnaA, CckA, LexA, CtrA, GafA/Dshi_1585, Dshi_3484/85) are located both at the *ori* and *ter* regions which could similarly couple expression of QS regulated traits to chromosome replication. The *divL* genes code for a histidine kinase and response regulator. Thus, a gradient of phosphorylation of CtrA during replication might be present which could be important for gene regulatory control.

As shown in the right panel, DNA is in a hemi-methylated state during replication, as the newly synthesized strain is not methylated yet. The conserved alphaproteobacterial DNA methyltransferase CcrM and transcription-factor GcrA (Brilli et al., 2010) have been shown to regulate expression of *ctrA* and other genes in *C. crescentus* in a methylation-state dependent way (Brilli et al., 2010; Fioravanti et al., 2013; Gonzalez et al., 2014). The *ori* and *ter* regions of *D. shibae* as well as the conjugative plasmids are enriched in the CcrM methylation-motif GANTC (Tomasch et al., 2018) which might influence replication based expression timing of genes located there.

Control of replication by CtrA has been covered by numerous articles (reviewed by Panis et al., 2015). When phosphorylated by CckA, *C. crescentus* CtrA represses replication and activates gene expression. Consequently, its phosphorylation state is tightly controlled throughout the cell cycle. Recently, c-di-GMP has been shown to switch the activity of CckA from kinase to phosphatase, releasing the *ori* for replication (Narayanan et al., 2018). If this regulatory connection is conserved in *D. shibae*, it might help to explain the observed shift in the proportion of replicating cells (Figure 4C). In response to the QS signal, more cells start to replicate and synthesize CtrA-phosphorelay components. When CckA accumulates at the cell poles its kinase activity is stimulated, as it has been observed in *C. crescentus*. The resulting increase in CtrA∼P inhibits the initiation of replication, consistent with the observed flow cytometry pattern change after 2 h. Simultaneously, CtrA∼P activates its target genes, resulting in differentiation into motile cells, GTA producing cells and cells that are able to replicate again. The possible CtrA dephosphorylating synthesis of c-di-GMP started after 2 h and increased to 300 pg/mg protein after 6 hurs when it still had not reached a plateau. The timing is consistent with the onset of replication in a larger subset of cells after the minimum has been reached at 6 h. LexA is upregulated last, probably due to the uptake of GTA DNA and probably enables the cell to integrate the acquired DNA into the chromosome and might also modulate expression of CtrA target genes.

The described timing of replication and trait expression controlled by CtrA∼P might also help to ensure that GTA packaging is prohibited in replicating cells. This would prevent a packaging bias toward the origin of replication. Expression dynamics of the GTA cluster showed two peaks, a fast but weak upregulation after 20–40 min, followed by a strong transcription of the complete gene cluster from 120 min onward (Figure 1E). This indicates that a GTA activating signal is already present in the early stage post AI induction and might be the AI itself or the CtrA phosphorelay activated by the AI, since at this stage, the fraction of replicating cells is increased. However, the threshold to fully activate GTA gene expression is reached after 2 h, when chromosome replication has almost stopped. A biphasic transcription pattern has also been detected for *Bartonella* GTAs (Québatte et al., 2017). In contrast to *D. shibae*, a strong early peak at the mid-exponential phase is followed by a weaker peak at the entry to stationary phase in *Bartonella*. However, uptake of GTA DNA and a simultaneous drop of population size due to the lysis of the GTA producing cells only takes place during the strong early peak in mid-exponential phase in *Bartonella*. In this group, the signal for the weaker peak at the entry to stationary phase is not known.

### Synthase LuxI_2_ Controls the Size of GTA Producing Subpopulation

We recently showed that GTA production in *D. shibae* is suppressed by LuxI_2_ (Tomasch et al., 2018). Here, we now used a combination of transcriptomics and Western blot examination to refine our understanding of GTA regulation. No GTA expression was observed in the QS null mutant Δ*luxI_1_* while a knockout of *luxI_2_* resulted in high GTA production as observed previously (Tomasch et al., 2018). A small amount of capsids was, however, produced in the Δ*luxI_1_* culture if it was stimulated by AI, and this was found for both concentrations of C14-oxo-HSL tested (500 nM and 2.5 μM), independent of the incubation time, and also for another AI, C18-dien-HSL, the main product of LuxI_1_. These data support our hypothesis, that the variability of GTA activation by QS is tightly controlled.

Recently, the direct activator of the GTA gene cluster, GafA, has been identified in *R. capsulatus* (Fogg, 2019). This gene is in turn directly activated by phosphorylated CtrA. Furthermore, the *gafA* promoter is bound by the AI-binding regulator GtaR. Therefore the author proposed that in *R. capsulatus* noise in the QS signal could control the size of the GTA producing subpopulation. GafA is conserved throughout the *Rhodobacteraceae* (Hynes et al., 2016) and present in *D. shibae* (Dshi_1584). As overexpression of GafA in *Ruegeria* also led to GTA overexpression, its regulatory role might be conserved, too. The proposed control of gafA and thereby GTAs through an AI-binding LuxR-type regulator is in accordance with our proposed QS-mediated limitation of GTA subpopulation size in *D. Shibae* (Figure 4A,C).

## Conclusion

Our proposed model helps to explain the observed timing of replication and gene expression. It remains to be elucidated how CtrA and c-di-GMP interact. The most interesting question remains how exactly AI synthase LuxI2 influences the size of the GTA producing subpopulation. A certain AI concentration or mixtures of different AIs might repress or activate GafA which then induces GTA gene cluster expression. Finally, our data suggest that key features of replication control, like gene localization or the role of c-di-GMP might be conserved among alphaproteobacteria.

## Author Contributions

JT and IW-D designed the study. SK performed experiments with help from HW. MS performed RNA sequencing. VK quantified c-di-GMP. SK and JT analyzed the data. SK wrote the manuscript with help from JT and IW-D.

## Conflict of Interest Statement

The authors declare that the research was conducted in the absence of any commercial or financial relationships that could be construed as a potential conflict of interest.

## Footnotes

1 https://github.com/ExpressionAnalysis/ea-utils

2 https://www.ncbi.nlm.nih.gov/geo/query/acc.cgi?acc=GSE122111
