# Supplementary material for: Integrated Transcriptional Regulatory Network of Quorum Sensing, Replication Control, and SOS Response in Dinoroseobacter shibae
Source: Front Microbiol. 2019 Apr 12;10:803. doi: 10.3389/fmicb.2019.00803 (PMC6473078; doi:10.3389/fmicb.2019.00803)
Supplement: Supplementary file 6 [file Data_Sheet_1.PDF]

Supplementary Material

Integrated transcriptional regulatory network of quorum sensing, replication control, and SOS response in *Dinoroseobacter shibae*

Sonja Koppenhöfer[1,2§*], Hui Wang[1], Maren Scharfe[3], Volkhard Kaever[4], Irene Wagner-Döbler[1], Jürgen Tomasch[1#*]

[1]*Group Microbial Communication, Technical University of Braunschweig, Braunschweig, Germany*

[2]*Institute for Chemistry and Biology of the Marine Environment (ICBM), University of Oldenburg, Oldenburg, Germany*

[3]*Group Genomic Analytics, Helmholtz Centre for Infection Research, Braunschweig, Germany*

[4]*Research Core Unit Metabolomics and Institute of Pharmacology, Hannover Medical School, Hannover, Germany*

[§]present address: Department of Biology, Memorial University of Newfoundland, St John's, NL, Canada

[#]present address: Department of Molecular Bacteriology, Helmholtz-Centre for Infection Research, Braunschweig, Germany

**Author for correspondence: skoppenhofer@mun.ca*

**Author for correspondence: Juergen.Tomasch@helmholtz-hzi.de*

This file contains:

Supplementary Figures S1-S11

Supplementary Tables can be found as separate excel-files

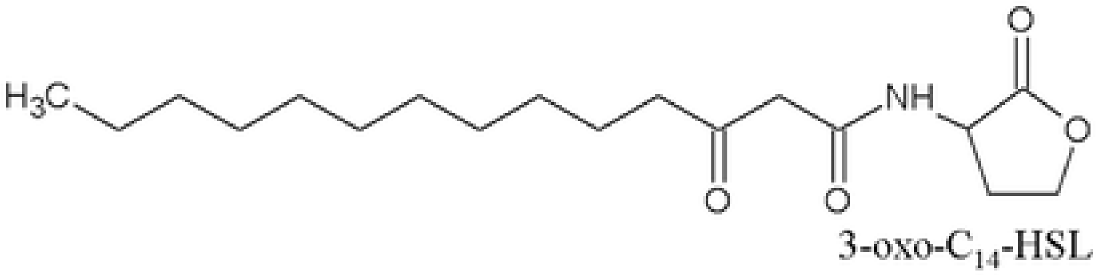

Supplementary Figure S1 – Structure of 3-oxo-C14-HSL used for QS induction experiments in this study.

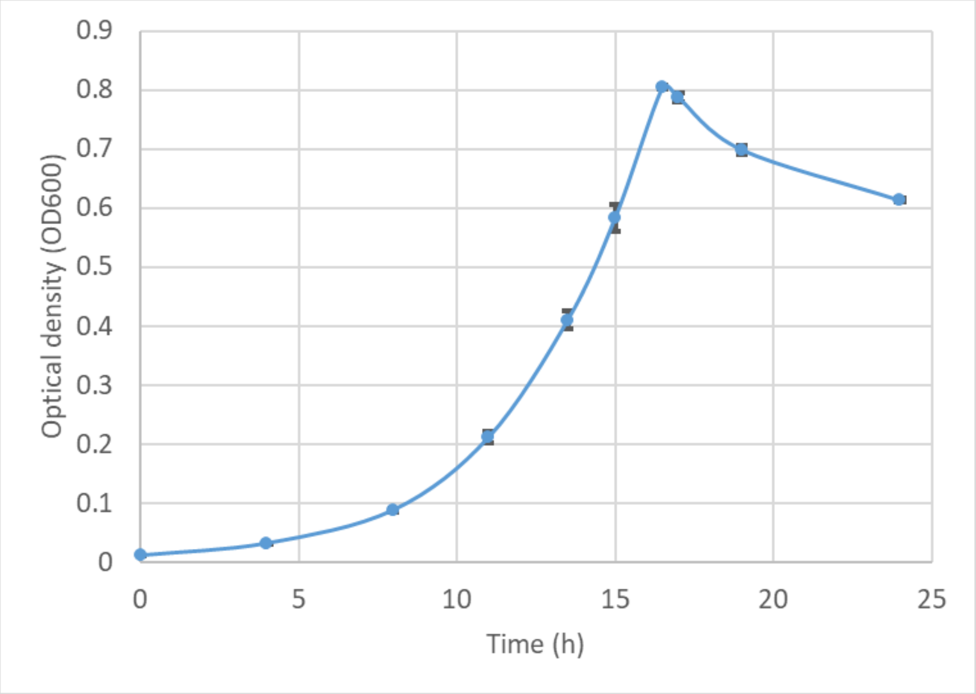

Supplementary Figure S2 – Growth of *D. shibae ΔluxI1* in artificial salt water medium. Three independent cultures were inoculated at OD600 0.01 and cultivated under the same conditions as RNAseq experiments were performed. Mean and standard deviation from three independent cultures are shown.

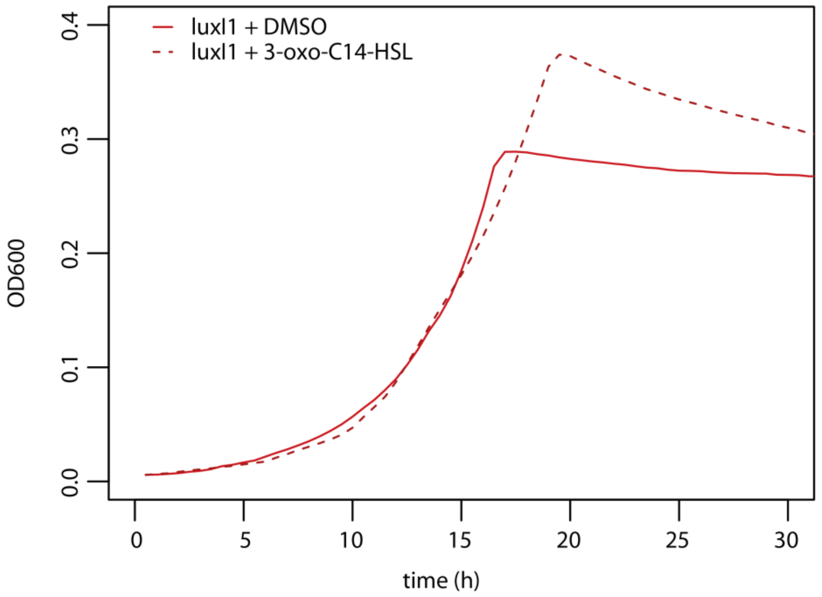

Supplementary Figure S3 – Growth of *D. shibae ΔluxI1* in artificial salt water medium with and without added AI. The curve represents the mean from 10 microtiter wells. Cell material from MB plates was inoculated into 20 ml of SWM medium and incubated overnight at 30°C and 160 rpm to obtain a pre-culture. Optical density at 600 nm (OD600) was measured with spectrophotometer Ultrospec 3100 pro (Biochrom Ltd, Cambridge CB4 0FJ England). Cultures were diluted to 0.01 OD600 in SWM medium and then 200 µl were placed into each well of a Honeycomb 2 plate (100 wells each plate, Oy Growth Curves Ab Ltd, Helsinki, Finland). OD600 was monitored every 30 min for 32 hours in the automated microbiology growth analysis system Bioscreen C (Oy Growth Curves Ab Ltd, Helsinki, Finland).

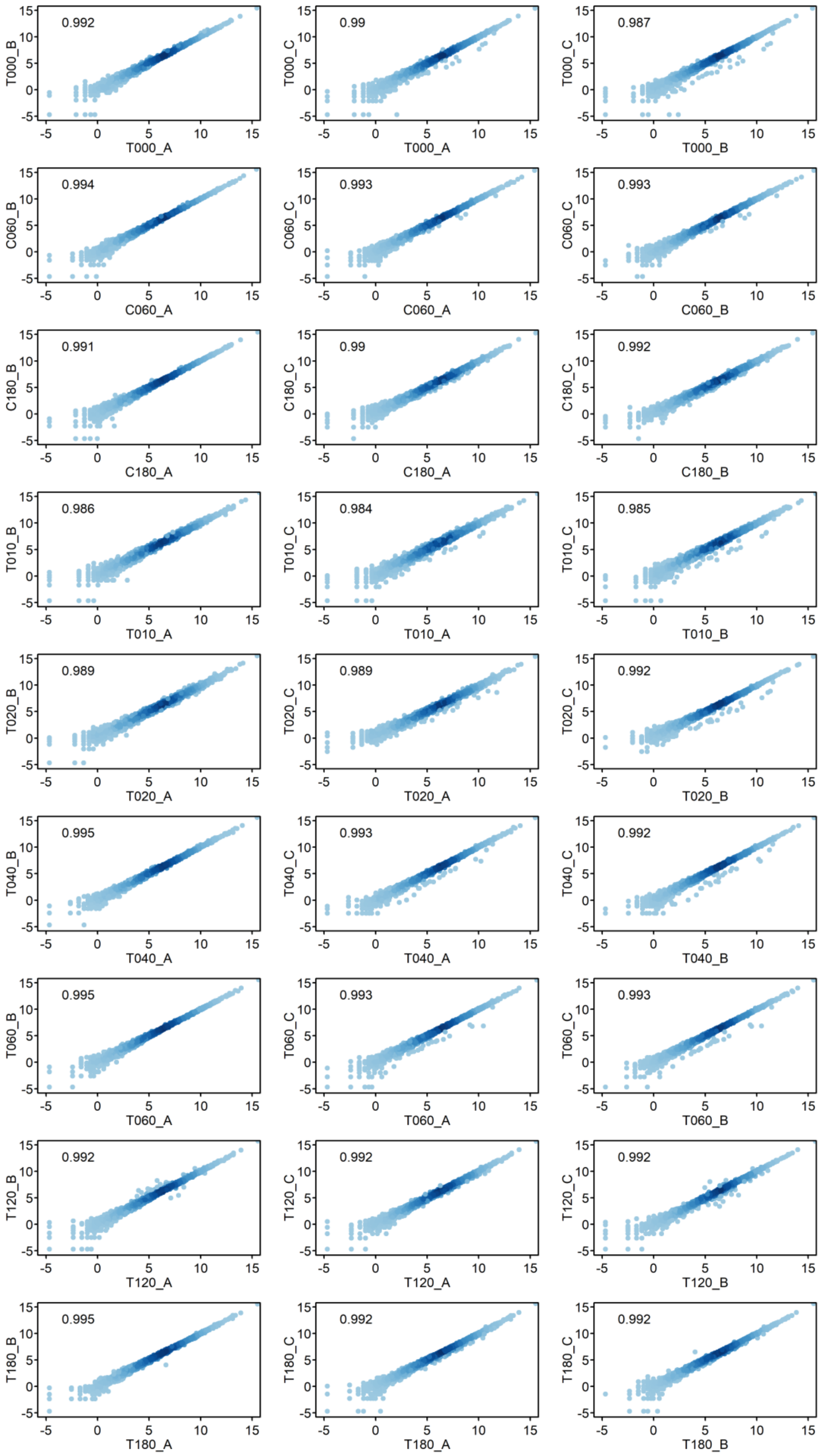

Supplementary Figure S4 – Correlation of replicate samples. Pairwise scatterplots of log_2 counts per million (CPM) normalized for differences in library size of the three replicates for one treatment are shown. Pearson correlation coefficient is shown in the upper left corner of the plot (Code used in sample names: C, control DMSO; T, treatment 3-oxo-C14 HSL; time is indicated in minutes followed by A-C as identifiers of the three replicates).

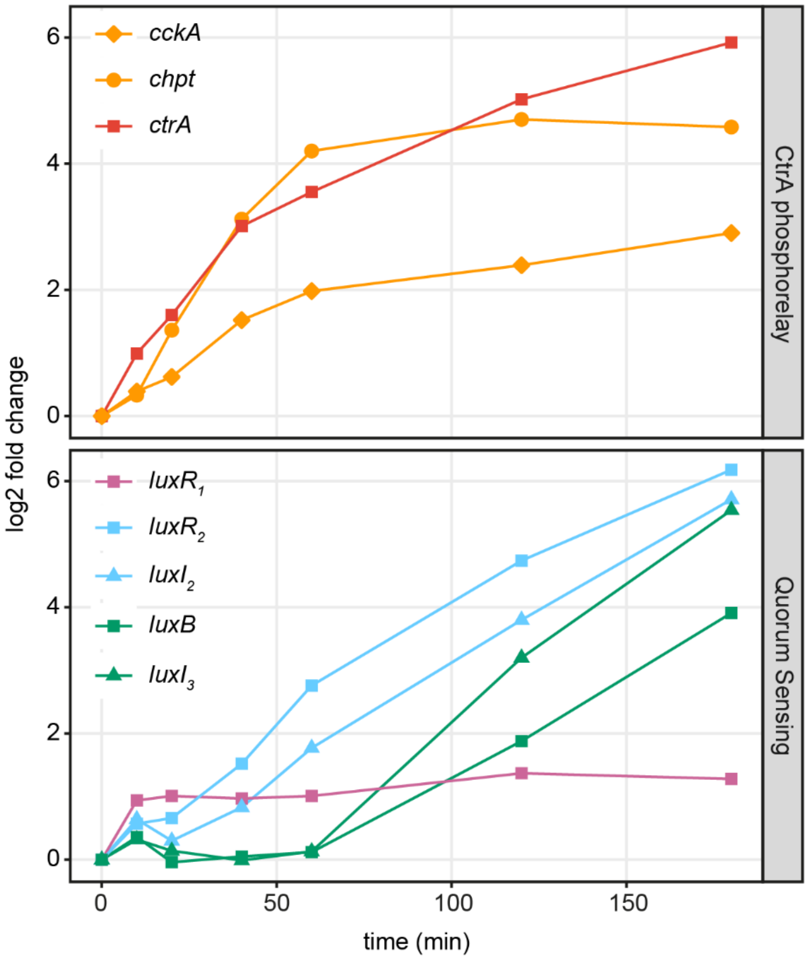

Supplementary Figure S5 – Time resolved transcriptional expression of the CtrA phosphorelay and Quorum sensing genes. Transcriptional expression (log2fold change) was observed until 180 min post-induction of Δ*luxI*$_1$ cells stimulated with 3-oxo C14 HSL. CtrA phosphorelay genes (histidine kinase *cckA*, phosphotransferase *chpT* and the transcriptional regulator *ctrA)* are displayed in the upper panel. The Quorum sensing synthases *luxI*$_2$ and *luxI*$_3$ and the regulators *luxR*$_1$*, R*$_2$ and *luxB* are shown in the lower panel.

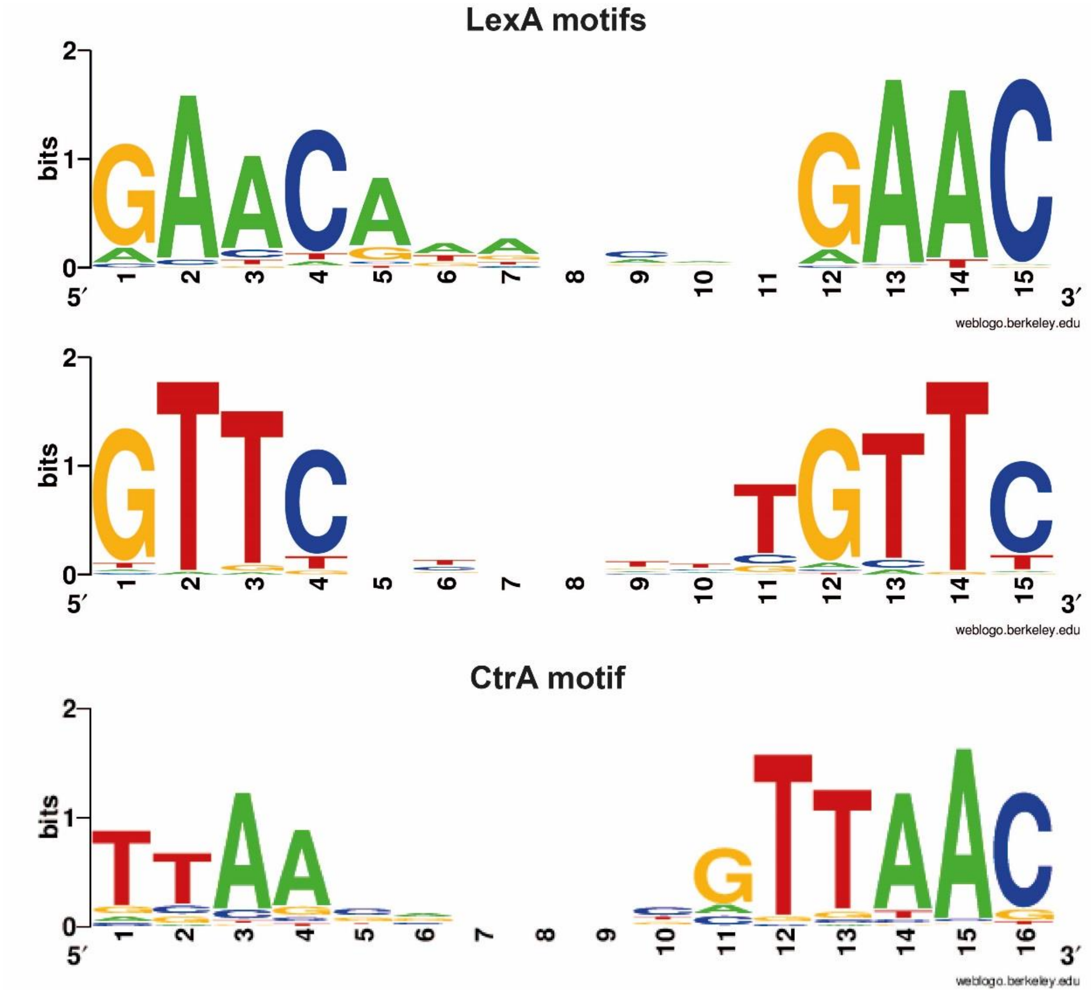

Supplementary Figure S6 – Sequence logo graphics of CtrA (A) and LexA (B). For *in silico* determination of CtrA we used the position weight matrix (PWM) determined for several *Alphaproteobacteria* (Brilli et al., 2010), and applied a minimum score of 82.5%. To determine LexA TFBS in *D. shibae* 80 *Alphaproteobacterial* sequences with in silico determined LexA binding site were downloaded (Erill et al., 2004). Based on these sequences a PWM was generated and searched for in the *D. shibae* genome. Genes with a minimum matching score of 82.5 % were kept and ordered to two groups, the GTTC consensus and GAAC consensus motifs.

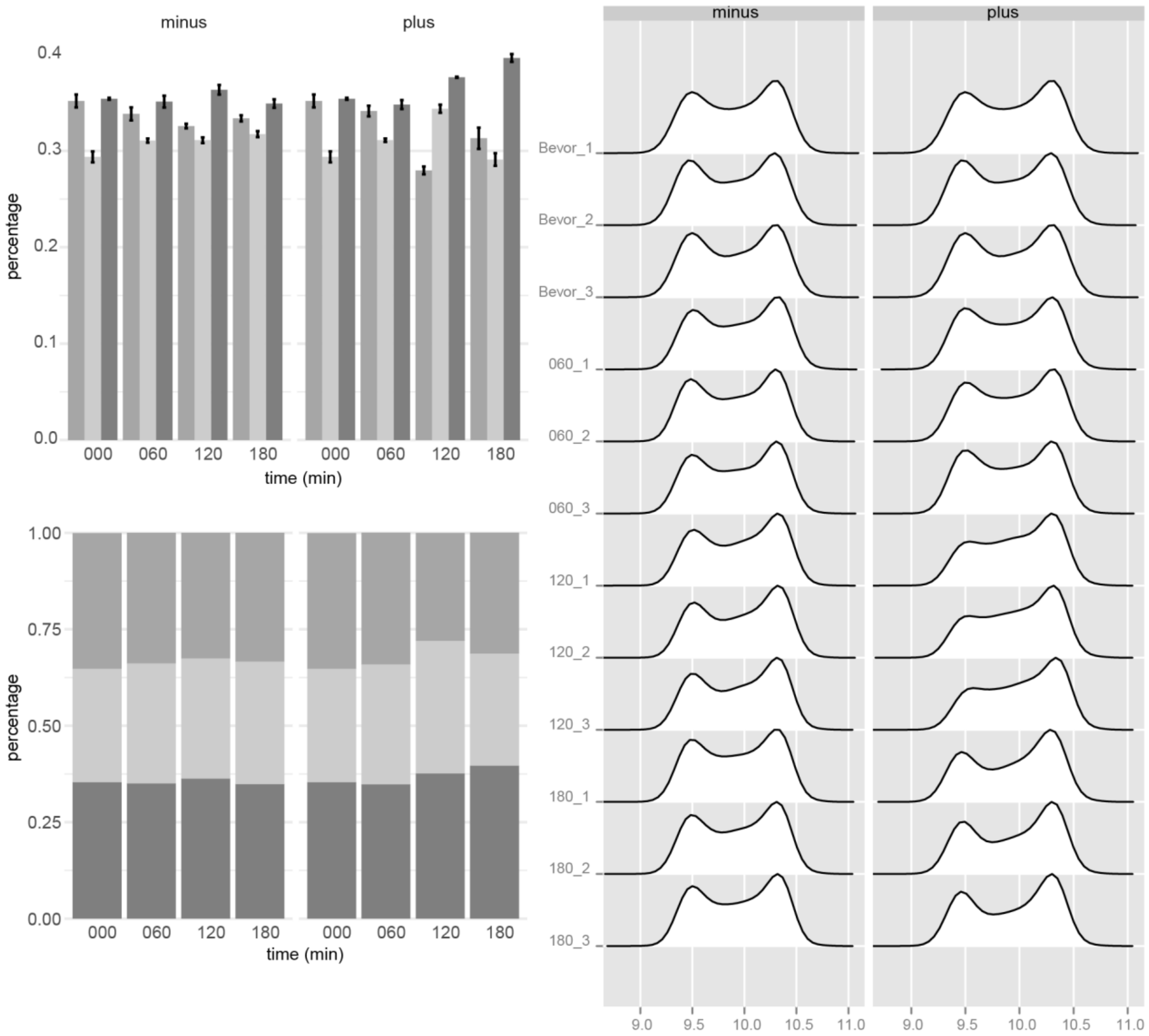

Supplementary Figure S7 – Three replicates are present for the flow cytometry experiments. (Left) Bar plot display of the three flow cytometrically determined fractions of one chromosome equivalent cells (dark grey), dividing cells (light grey) and cells with two chromosome equivalents (medium grey) in the population in percentage over a period of 180 min post induction with either DMSO as negative controle (minus) or 3-oxo C14 HSL (plus).

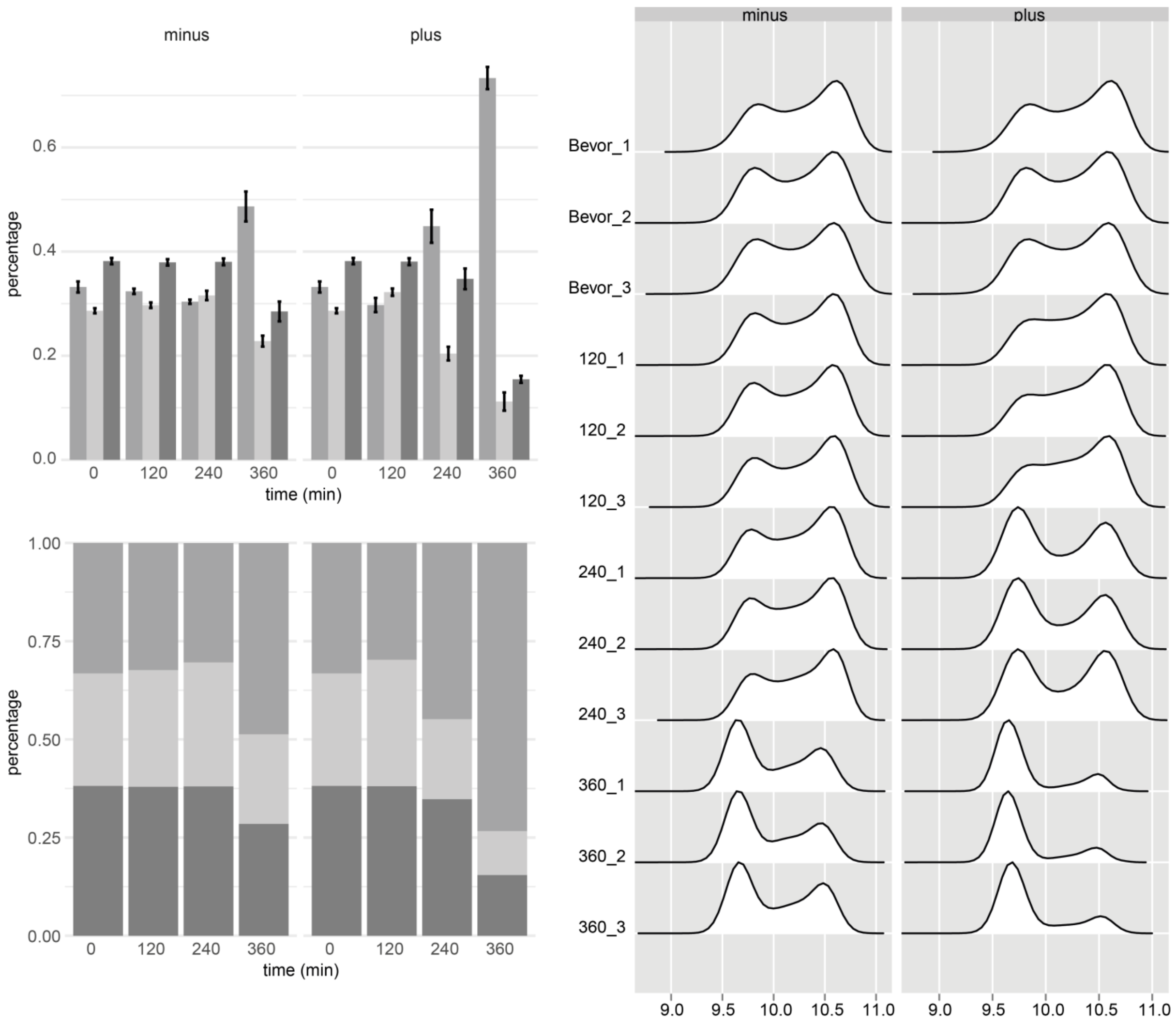

Supplementary Figure S8 – First reproduction of the flow cytometry experiment, run over a period of 360 min post induction.

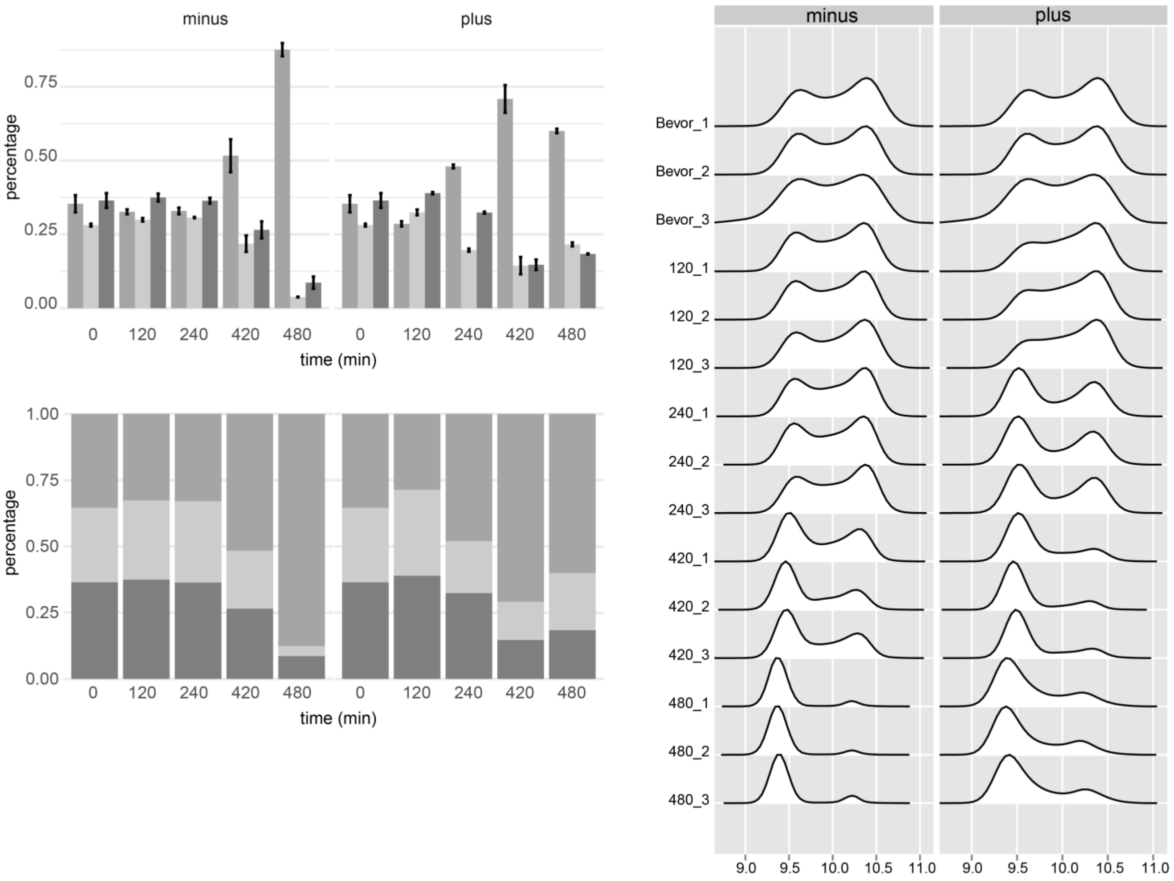

Supplementary Figure S9 – Second reproduction of the flow cytometry experiment, run over a period of 480 min post induction.

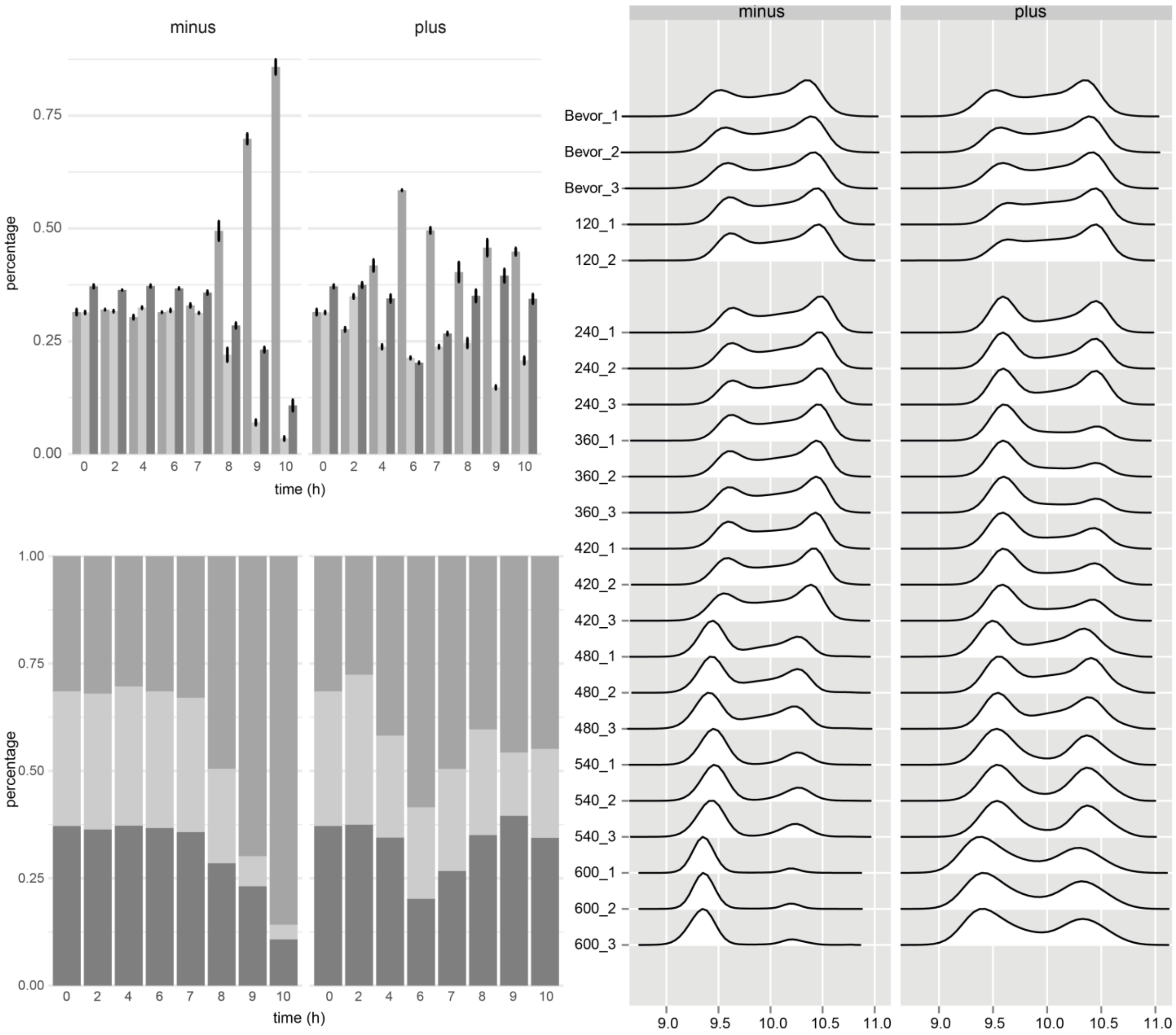

Supplementary Figure S10 – Third reproduction of the flow cytometry experiment, run over a period of 600 min post induction.

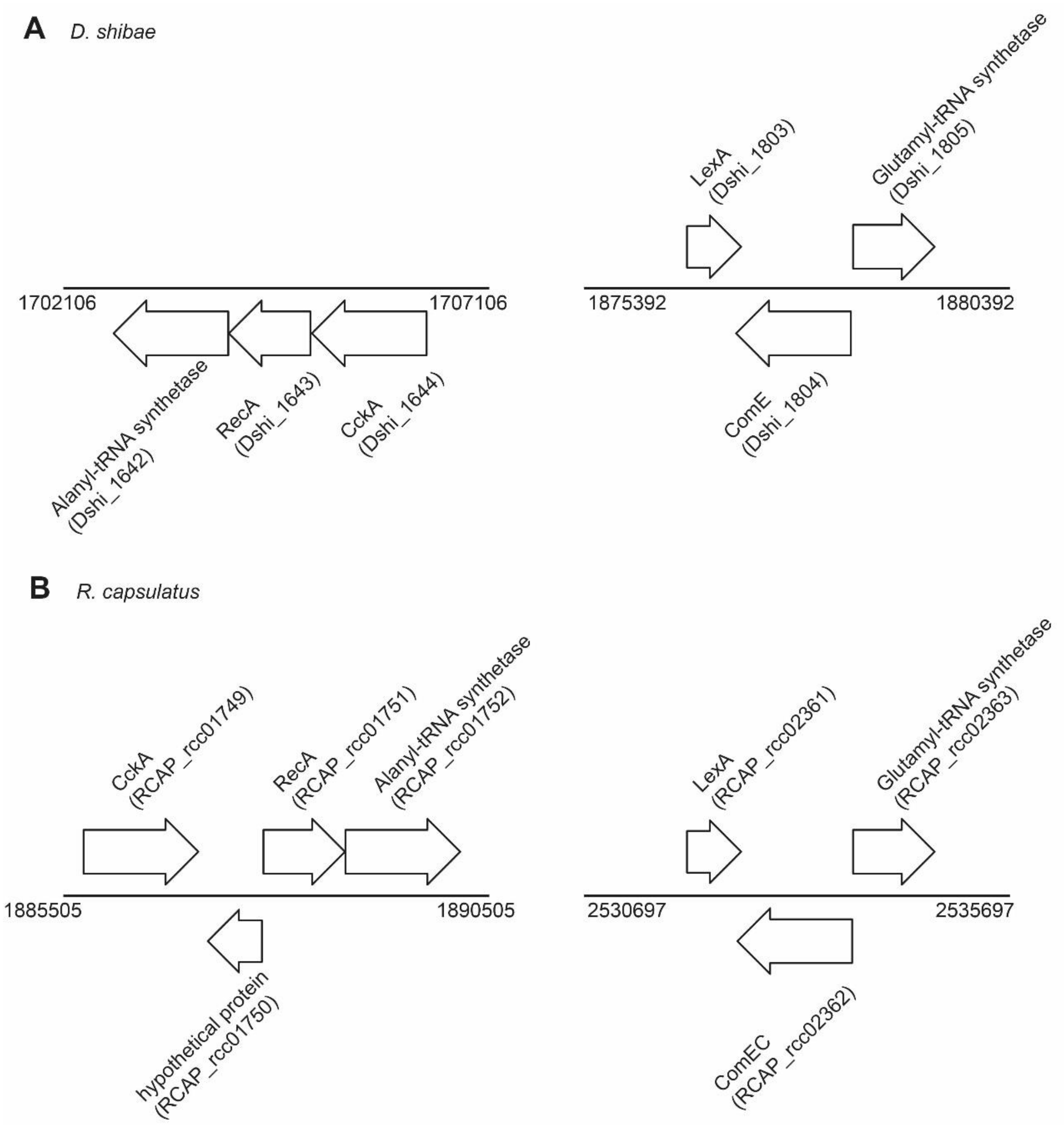

Supplementary Figure S11 – Comparison of gene location in *D. shibae* and *R. capsulatus*. Both organisms display the co-location of *cckA, recA* and *alanyl-tRNA synthase* (interrupted by a rcc01750 in *R. capsulatus*) as well as *lexA, comE*(*C*) and *glutamyl-tRNA synthetase*in both organisms. The last succession is even oriented on the same strand.
